# Baseline thrombospondin-1 concentrations are not associated with mortality in septic patients: a single-center cohort study on the intensive care unit

**DOI:** 10.1186/s40635-017-0120-y

**Published:** 2017-01-25

**Authors:** Ruben J. van der Wekken, Hans Kemperman, Mark Roest, Dylan W. de Lange

**Affiliations:** 1 Intensive Care Department, University Medical Center Utrecht, Room F06.149, 3508 GA Utrecht, the Netherlands; 2 Department of Clinical Chemistry and Haematology, University Medical Center, University Utrecht, Utrecht, the Netherlands; 3 Synapse B.V., Maastricht, the Netherlands; 4 Intensive Care Department, University Medical Center Utrecht, University Utrecht, Utrecht, the Netherlands

**Keywords:** Thrombospondin-1, Biomarker, Sepsis, Outcome, Mortality, Intensive care unit

## Abstract

**Background:**

The initial phase of sepsis is characterized by hyperinflammation. Levels of thrombospondin-1 (TSP-1) rise rapidly during acute inflammation. The purpose of this clinical study was to study the association between plasma TSP-1 levels and mortality in patients with sepsis on the intensive care unit.

**Methods:**

Critically ill adult patients with sepsis, severe sepsis, or septic shock were included. They were further divided into tertiles based on their baseline plasma TSP-1 concentrations. Primary outcome measure was 28-day mortality. Furthermore, associations with severity of sepsis and platelet counts were studied.

**Results:**

Two hundred thirty-five patients were included. Median plasma TSP-1 concentrations of the tertiles were 194, 463 and 874 ng/mL, respectively. There were no baseline differences. Mortality rates (26.6, 16.7, and 16.7%, *p* = 0.20) and cumulative survival curves (*p* = 0.22) were not statistically different between the tertiles. There was no association of baseline TSP-1 with severity of sepsis (*p* = 0.08). TSP-1 and platelet counts were positively correlated (159, 198, and 295 × 10^9^/L, *p* = 0.04).

**Conclusions:**

Baseline plasma levels of TSP-1 were not associated with mortality and severity of sepsis in mixed population of septic ICU patients. Further research is needed to clarify the expression of TSP-1 and to unravel the potential prognostic value of this biomarker in human sepsis.

**Electronic supplementary material:**

The online version of this article (doi:10.1186/s40635-017-0120-y) contains supplementary material, which is available to authorized users.

## Background

Despite improvements in intensive care medicine, sepsis still has an unacceptably high mortality rate (18–36%) [1]. Swift recognition of sepsis and proper distinction of the clinical symptoms from non-infectious inflammation are crucial. Sepsis should be treated promptly with antibiotics while inflammation does not benefit from this treatment. In both sepsis and inflammation, the immune system plays an important role. Its response can be characterized by hyperinflammation initially, with a subsequent immunosuppressive phase. Immunomodulatory proteins could therefore serve as biomarkers of both inflammation and sepsis.

Thrombospondin-1 (TSP-1) is a glycoprotein which is mainly found in the alpha granules of platelets but is also secreted by many other cells, including endothelial cells, leukocytes, smooth muscle cells, monocytes, and macrophages, upon stimulation by cytokines, growth factors, and stress [2–5]. After secretion, it binds with proteins on the cell membrane and extracellular matrix and is involved in endothelial cell adhesion, motility, and growth, contributing to platelet aggregation, angiogenesis, wound healing, and the immune response [6, 7].

During acute inflammation, TSP-1 is transiently released and has various effects on the immune system: it activates transforming growth factor β1 (TGF-β1), induces intense chemotaxis, negatively regulates T cell activation, induces apoptosis in endothelial cells, activates neutrophils, and regulates nitric oxide (NO) influencing vasodilatation and chemotaxis [4, 8–11]. TSP-1 was identified as an element of gene expression signatures predictive of poor outcome in pediatric sepsis and in trauma patients [12, 13]. Increased expression of TSP-1 on platelets in sepsis presumably contributes to sequestration of platelets and thus the development of the multiple organ dysfunction syndrome [14]. In several animal studies, TSP-1-deficient mice showed increased survival in *Escherichia coli* peritoneal sepsis, systemic candidiasis, and Klebsiella pneumonia, compared to wild type mice [15–17]. Therefore, high levels of TSP-1 could be associated with poorer outcome in human sepsis. To our knowledge, the potential prognostic value of this biomarker has not been studied in human sepsis. Our hypothesis was that higher levels of TSP-1 were associated with poorer outcome in septic intensive care unit (ICU) patients. We therefore conducted an analysis on a mixed cohort of septic patients in the ICU.

## Methods

### Study design and selection criteria

This cohort study is a retrospective analysis of prospectively collected data in a 6-month period in 2009 in the ICU of the University Medical Center of the University of Utrecht (UMCU) in the Netherlands, an academic hospital with 1042 beds. Our mixed ICU receives admissions from all specialties (surgical, medical, transplant, cardiosurgical, neurosurgical, trauma, etc.) except burns. The entire ICU consists of 30 beds and receives over 2200 admissions annually.

The minimum age for inclusion was 18 years. Patients were included whenever they had a clinical suspicion of sepsis. Sepsis was defined as a combination of a systemic inflammatory response syndrome (SIRS) on admission and a suspicion of having infection. SIRS means two or more of the following symptoms: fever (>38.0 °C or <36.0 °C), white blood cell count (>12 × 10^9^/L or <4 × 10^9^/L), tachypnoea (respiratory rate >20/min or P_a_CO_2_ <4.3 kPa), or tachycardia (heart frequency >90 beats per minute). However, patients who were anticipated to stay less than 24 hours in the ICU (e.g., planned, uncomplicated surgical patients) were excluded.

The primary outcome measure was 28-day mortality. The secondary outcome measure was severity of sepsis. Furthermore, the association of baseline TSP-1 levels with platelet counts and the use of antiplatelet drugs and heparins were studied.

### Procedures and definitions

During the period of research, each newly admitted patient in the ICU was either in- or excluded based on the abovementioned criteria. Furthermore, patients developing SIRS and being suspected of having an infection while in the ICU were also included. At the time of study inclusion, blood was withdrawn for analysis. On a daily basis, research nurses collected the patient data (like maximum temperature, heart rate, leukocyte count, etc.) during a maximum period of 10 days or until discharge or death. TSP-1 values were not part of the normal clinical routine and were not made available for attending physicians during the study, and therefore, they did not influence decision making.

Levels of TSP-1 were measured using a semiautomated ELISA on a Tecan Freedom EVO robot (Tecan, Switzerland). First, blood was collected in heparin tubes. Before thawing, 3.2% citrate solution was added in a 1:9 solution:plasma ratio. Samples were diluted by the Tecan Freedom EVO ELISA robot, using 1% bovine plasma albumin in phosphate-buffered salt. Captured antibodies R&D mouse antihuman thrombospondin-1 [Catalog DY3074] (0.5 ng/mL) and R&D goat antihuman thrombospondin-1; biotin-labelled [Catalog DY3074] (100 ng/mL) DAKO streptavidin; and HRP-coupled antibody [Catalog P0379] (0.71 μg/mL) were coated overnight at 4 °C. Antigens were measured on separate 384 well Nunc MaxiSorp ELISA plates (Nunc, Denmark). The procedure is described more in detail in another study [18].

The Centers of Disease Control have published algorithms for “proven infection” of health care-associated infection and criteria for specific types of infections in the acute care setting [19]. We adhered to these definitions to classify “possible” versus “proven” infection. The definitions of sepsis, severe sepsis, and septic shock were in agreement with a previous publication [20], although an infection did not need to be proven; suspicion of infection was enough. Patients, who turned out to have no infection, were subsequently excluded from the analysis.

### Statistical analysis

Categorical variables are presented as counts with proportions and continuous variables as means (with standard deviation) or as medians (with interquartile range), based on the normality of distribution, determined by the Kolmogorov–Smirnov test. Comparisons between tertiles of TSP-1 were made using Pearson’s chi-square test for categorical variables and with the analysis of variance or the non-parametric Kruskal–Wallis test for continuous variables. The cumulative survival was calculated by applying the Kaplan–Meier method, and differences in mortality were compared with the log-rank test. A two-tailed *p* < 0.05 was considered to indicate statistical significance. Receiver operating characteristic (ROC) curve is presented, with its area under the curve (AUC) and 95% confidence interval.

All statistical analyses were performed with SPSS Statistics 21.0 (SPSS Inc., Chicago, IL, USA).

## Results

### Patient characteristics

Of the 275 patients in the cohort study [18], 30 patients were excluded because they were not suspected of having an infection; nine patients were excluded because the TSP-1 measurements were missing, and one patient with an outlying TSP-1 level on day 1 was excluded. In total, 235 could be included for analysis. The patients were divided into tertiles based on their baseline TSP-1 level. There were no significant differences in clinical characteristics between the patient groups (Table 1). The distributions of C-reactive protein (CRP), procalcitonin (PCT) levels, and APACHE IV scores were not statistically different between the three tertiles of TSP-1.

**Table 1:** Clinical characteristics according to tertiles of baseline thrombospondin-1 concentration

	Tertile 1	Tertile 2	Tertile 3	*p*
*n* (%)	79 (33.6)	78 (33.2)	78 (33.2)	
TSP-1 (ng/mL), median (IQR^b^)	194 (102–258)	463(399–571)	874 (765–1174)	
Men, *n* (%)	57 (72.2)	50 (64.1)	44 (56.4)	0.12
Age (years), median (IQR)	65 (52–75)	60 (45–70)	62 (52–74)	0.11
BMI (kg/m2), median (IQR)	24.5 (22.6–27.0)	24.8 (22.0–27.0)	23.9 (22.0–27.8)	0.97
Admission via OR, *n* (%)	20 (25.3)	18 (23.1)	20 (25.3)	0.95
APACHE IV (score), median (IQR)	74 (54–95)	81 (60–97)	72 (55–86)	0.56
CRP (mg/L), median (IQR)	172 (90–285)	215 (145–298)	200 (127–299)	0.19
PCT (ug/L), median (IQR)	1.69 (0.49–7.19)	1.45 (0.37–7.58)	1.82 (0.64–5.07)	0.89
Severity of sepsis				
Sepsis, *n* (%)	43 (31.6)	54 (39.7)	39 (28.7)	0.08
Severe sepsis, *n* (%)	15 (30.6)	12 (24.5)	22 (44.9)	
Septic shock, *n* (%)	21 (42)	12 (24)	17 (34)	

Data are presented as numbers with proportions and medians with IQR (TSP-1, age, BMI, APACHE IV score, CRP, and PCT are not normally distributed)

*TSP-1* thrombospondin-1, *IQR* interquartile range, *BMI* body mass index, *OR* operation room, *APACHE* Acute Physiology and Chronic Health Evaluation, *CRP* c-reactive protein, *PCT* procalcitonin

### Mortality

**Fig. 1:**
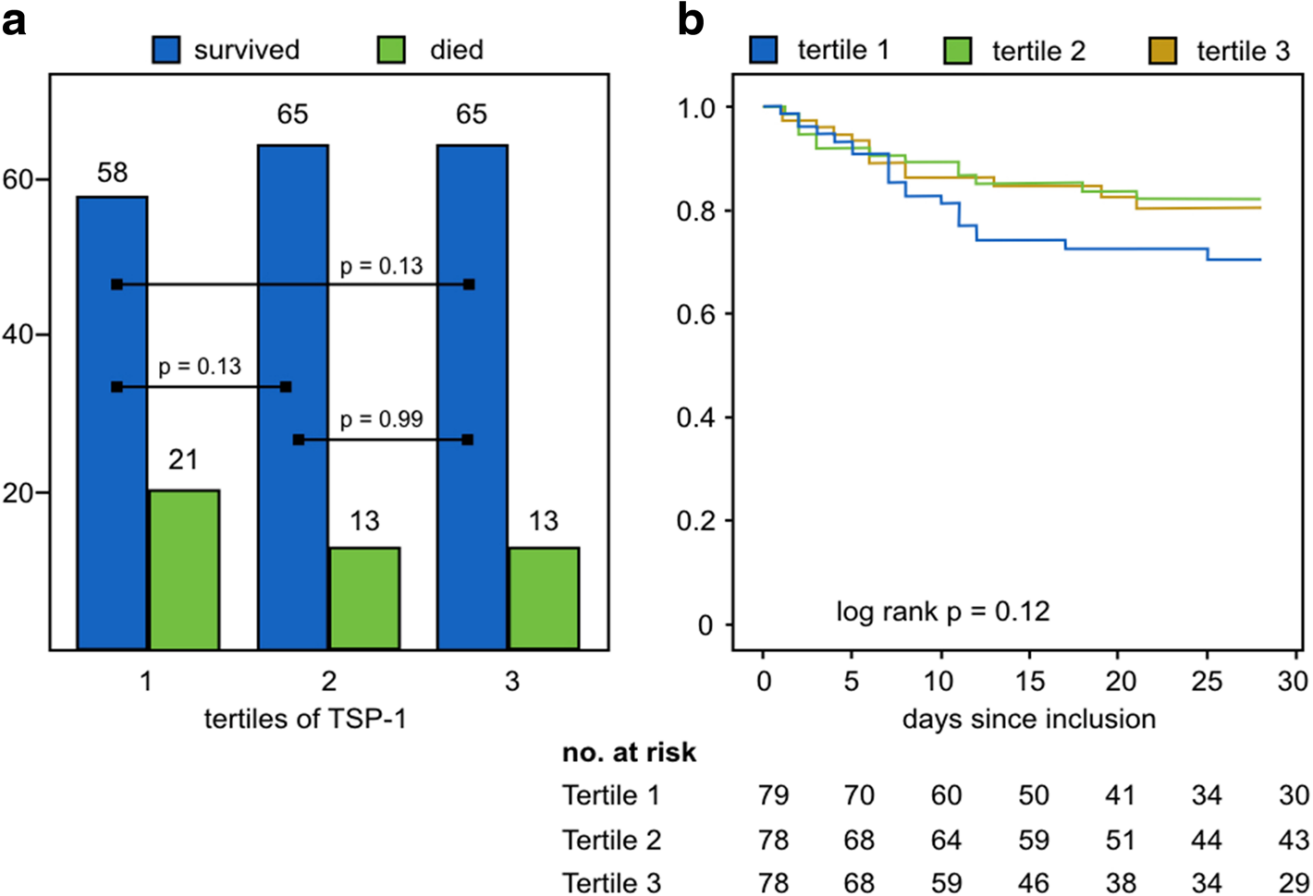
**a** Mortality (*absolute numbers*) in different tertiles of TSP-1 do not differ statistically. **b** Kaplan–Meier 28-day survival plots for the three tertiles, including number of patients at risk

Of the 235 patients, 47 (20.0%) patients died within 28 days. In tertile 1, the mortality rate during the study period was 26.6% (*n* = 21), in tertile 2 16.7% (*n* = 13), and in tertile 3 16.7% (*n* = 13), which was not statistically significant (*p* = 0.20). Survival analysis showed no differences in cumulative survival (*p* = 0.22) (Fig. 1).

**Fig. 2:**
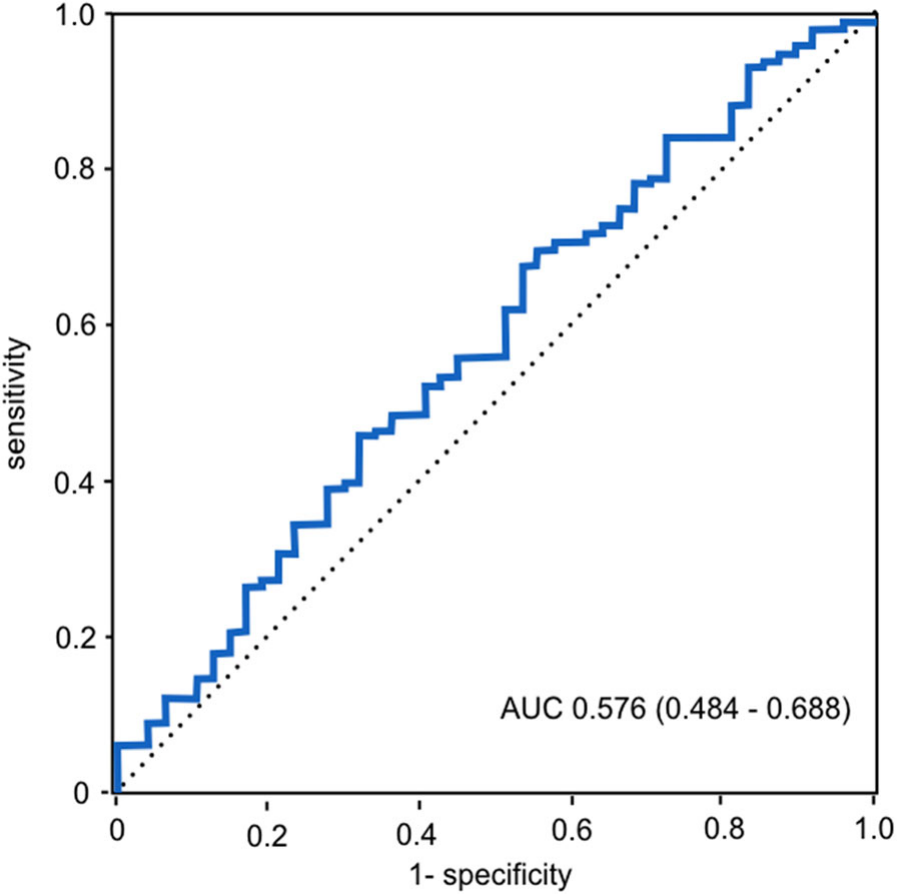
Receiver operating curve for TSP-1 and 28-day mortality

In Fig. 2, the ROC curve is presented, showing the ability of TSP-1 to predict 28-day mortality, with AUC of 0.58 (95% confidence interval, 0.48–0.67).

### Secondary outcomes

**Fig. 3:**
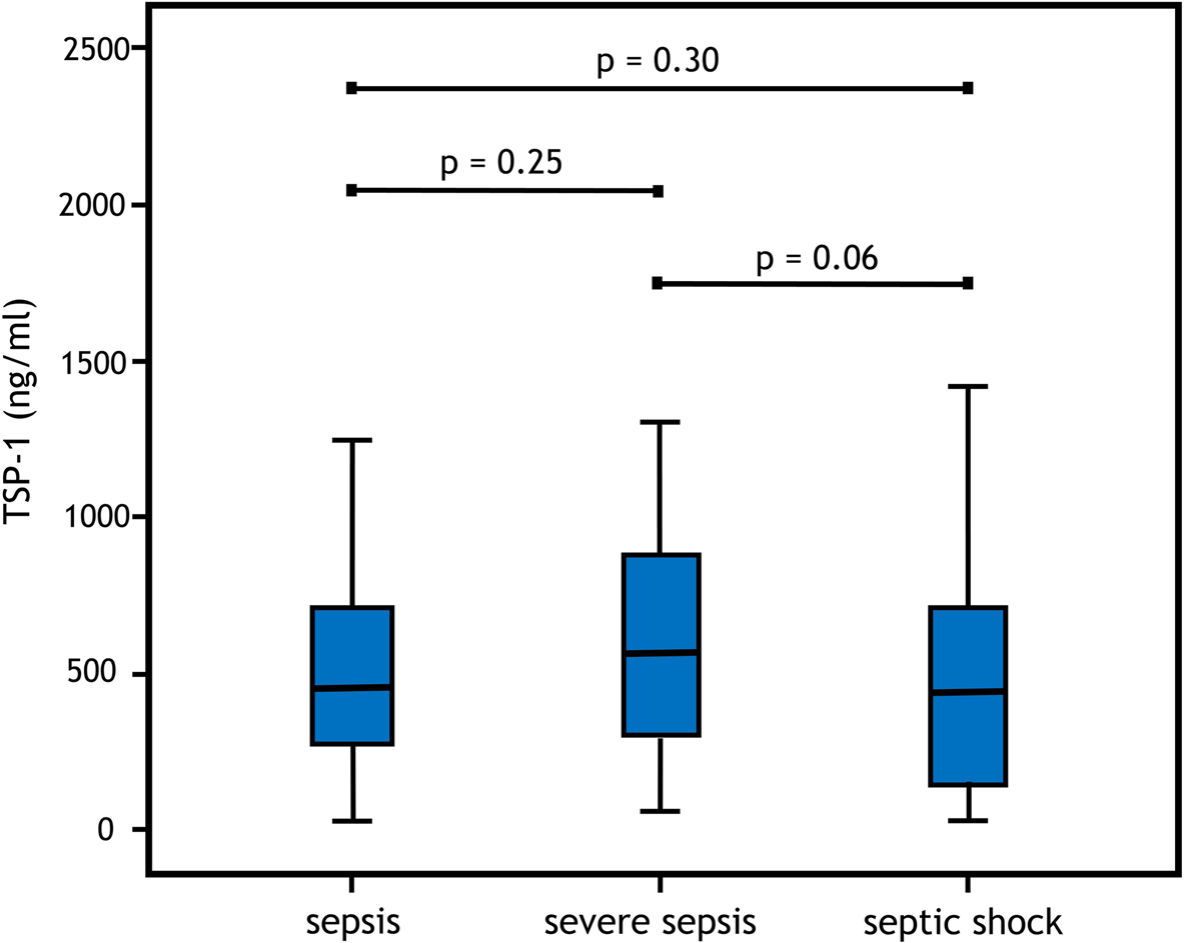
Median levels of baseline TSP-1 plasma levels in sepsis, severe sepsis, and septic shock

In the sepsis group (*n* = 136), there was a mortality rate of 16.2% (*n* = 22), in the severe sepsis (*n* = 49) 16.3% (*n* = 8), and the septic shock (*n* = 50) 34.0% (*n* = 17, *p* = 0.02). Median concentrations of baseline TSP-1 were not significantly different between patients with sepsis, severe sepsis, and septic shock. In Fig. 3, the interquartile ranges are shown. Within the tertiles, severity of sepsis was equally distributed (Table 1).

**Fig. 4:**
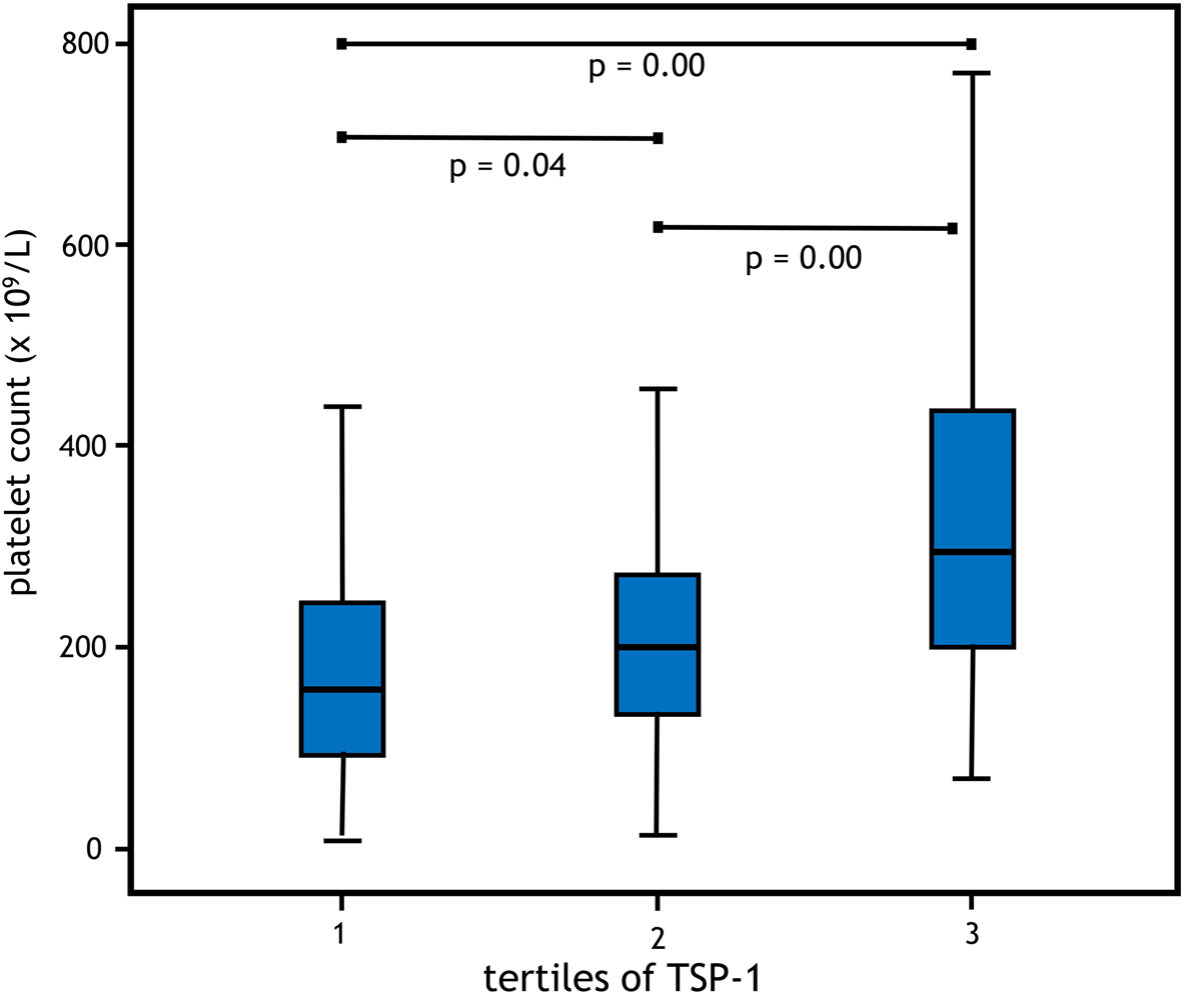
Median platelet counts in the different tertiles of TSP-1

There were statistically significant differences in platelet counts and thrombocytopenia (<150 × 10^9^/L) between the TSP-1 tertiles (Fig. 4). Thrombocytopenia (*n* = 70), analyzed in the total cohort, was associated with mortality (33% versus 14%, *p* < 0.01). The use of acetylsalicylic acid (ASA), low molecular weight heparin (LMWH), and unfractionated heparin (UFH) was not different between the tertiles, as shown in Table 2.

**Table 2:** Platelets and use of antiplatelet drugs and heparin in the tertiles

		Tertile 1	Tertile 2	Tertile 3	*p*
Platelet count (×10^9^/L), median (IQR)		159 (93–245)	198 (137–272)	295 (201–438)	0.04
Thrombocytopenia (<150 × 10^9^/L), *n* (%)		35 (47)	23 (32)	12 (16)	0.00
ASA use, *n* (%)		12 (15)	14 (18)	16 (21)	0.68
Heparin, *n* (%)	None	8 (10)	4 (5)	5 (6)	0.35
	LMWH, prophylactic	53 (67)	49 (64)	58 (74)	
	LMWH, therapeutic	12 (15)	20 (26)	10 (13)	
	UFH	6 (8)	4 (5)	5 (6)	

Data are presented as numbers with proportions and medians with IQR (platelet counts are not normally distributed)

*ASA* acetylsalicylic acid, *LMWH* low molecular weight heparin, *UFH* unfractionated heparin

## Discussion

The purpose of this clinical study was to study the association between TSP-1 levels and mortality within 28 days in ICU patients with sepsis. Patients were divided into tertiles based upon their plasma TSP-1 level at baseline. Values in the lowest tertile (median, IQR 194, 102–258 ng/mL) were comparable with the range found in healthy humans (40–250 ng/mL). [21]. In this study, no association between TSP-1 levels and mortality was found.

There are several hypotheses why high TSP-1 levels are associated with poor outcome. TSP-1 is secreted upon acute inflammation, cytokine release, and stress. Furthermore, several comorbidities, such as diabetes, chronic renal failure, chronic liver failure, and acute myocardial infarction, are associated with higher TSP-1 levels [21, 22]. Moreover, in animal studies, TSP-1-deficient mice were found to have lower mortality rates in *E. coli* peritoneal sepsis, systemic candidiasis, and Klebsiella pneumonia, and TSP-1 seemed to inhibit inflammatory leukocytes [15–17]. However, TSP-1-deficient mice might not be comparable with clinical patients with low plasma levels, given the extensive binding capacity of TSP-1 and its concomitant multifunctional nature [23]. On the other hand, TSP-1-deficient mice were demonstrated to have an only slightly different phenotype than their control group, possibly due to compensation by genetic adaption [24, 25]. In two of these mice studies, the results were explained by inhibition of macrophages by TSP-1 [15, 16]. However, this inhibition was in vitro, whereas in vivo TSP-1 was found to promote phagocytosis in atherosclerotic lesions by macrophages and to mediate increased phagocytosis during hypoxia [26, 27]. Caution should be taken in extrapolating in vitro observations to in vivo situations, because the influence of TSP-1 on the inflammation response is complex and depends on multiple factors, including peroxisome proliferator-activated receptor expression on leukocytes [4] and the expression of CD36 and CD47. Both pro- and anti-inflammatory activities of TSP-1 have been described, and the effect of TSP-1 seems to be context-related and cofactor-dependent.

Vice versa, there are also reasons to assume that lower TSP-1 levels are associated with poor outcome. Platelets are the main source of TSP-1 secretion, and thrombocytopenia was associated with increased 28-day mortality [28]. Therefore, thrombocytopenia might lead to lower levels of TSP-1 and yet to higher mortality. Indeed, in the present study, in the highest TSP-1 tertile, the median platelet counts were higher and the incidence of thrombocytopenia was significantly lower than in the other tertiles. However, the relationship with lower levels of TSP-1 (despite lower platelet counts) and mortality could not be decerned in this study population.

The use of unfractioned heparin and low molecular weight heparin is associated with decreased levels of TSP-1 [29, 30]. Aspirin therapy, however, did not influence plasma levels of TSP-1 in women with breast cancer [31]. We could not find a significant difference in the use of such medications between the three tertiles. Therefore, the use of these medications was not a definite confounder in the relationship between TSP-1 and outcome.

A possible explanation for our results is that patients were in different phases of their course of sepsis at study inclusion. TSP-1 at inclusion of the study do not necessarily reflect TSP-1 levels at the start of infection. This might also explain why other biomarkers, such as CRP and PCT, were not different between the tertiles. There are several limitations that need to be addressed. First, our study population is heterogeneous in source and severity of sepsis. For example, in surgical patients, TSP-1 is known to be increased particularly in the first 24 hours after incisional wounds [32]. However, the numbers of surgical patients in the tertiles were comparable. On the other hand, in a mouse cecal ligation and puncture model, TSP-1 was found to rise in the first 2 hours and remained high for 72 hours [33]. Second, tissue levels of TSP-1 rise while aging [34]; however, demographic characteristics like age were not different at baseline. Third, the fact that ICU sepsis is a clinical diagnosis could cause sampling bias, compromising the external validity of this study. Fourth, the use of suspicion of infection as inclusion criterion could dilute the discriminative effect of TSP-1, because patients who turn out to have no infection will be excluded, whereas people with an infection could have been missed. Last, as with many cohort studies in the ICU, mortality is not always the direct consequence of the initial problem.

Future research should explore the expression of TSP-1 in human sepsis, while focusing on the influence of pre-sepsis TSP-1 concentrations, its trend during the course of sepsis, and its association with outcome.

## Conclusions

In conclusion, baseline plasma levels of TSP-1 were not associated with mortality and severity of sepsis in mixed population of septic ICU patients. Further research is needed to clarify the expression of TSP-1 and unravel the potential prognostic value of TSP-1 in human sepsis.

## Additional file

Additional file 1: Dataset. (XLSX 29 kb)

## Abbreviations

ICU: Intensive care unit
NO: Nitric oxide
SIRS: Systemic inflammatory response syndrome
TGF-β1: Transforming growth factor β1
TSP-1: Thrombospondin-1
UMCU: University Medical Center of the University of Utrecht
